# Accelerated long-term forgetting in healthy older adults predicts cognitive decline over 1 year

**DOI:** 10.1186/s13195-020-00693-4

**Published:** 2020-09-28

**Authors:** Alfie R. Wearn, Esther Saunders-Jennings, Volkan Nurdal, Emma Hadley, Michael J. Knight, Margaret Newson, Risto A. Kauppinen, Elizabeth J. Coulthard

**Affiliations:** 1 Bristol Medical School, University of Bristol, Bristol, UK; 2 Institute of Clinical Neurosciences, North Bristol NHS Trust, Bristol, UK; 3 Department of Psychology, University of Bath, Bath, UK; 4 School of Psychological Science, University of Bristol, Bristol, UK

**Keywords:** Long-term memory, MRI, Hippocampus, Medial temporal lobe, Alzheimer’s disease, Early diagnosis

## Abstract

**Background:**

Here, we address a pivotal factor in Alzheimer’s prevention—identifying those at risk early, when dementia can still be avoided. Recent research highlights an accelerated forgetting phenotype as a risk factor for Alzheimer’s disease. We hypothesized that delayed recall over 4 weeks would predict cognitive decline over 1 year better than 30-min delayed recall, the current gold standard for detecting episodic memory problems which could be an early clinical manifestation of incipient Alzheimer’s disease. We also expected hippocampal subfield volumes to improve predictive accuracy.

**Methods:**

Forty-six cognitively healthy older people (mean age 70.7 ± 7.97, 21/46 female), recruited from databases such as Join Dementia Research, or a local database of volunteers, performed 3 memory tasks on which delayed recall was tested after 30 min and 4 weeks, as well as Addenbrooke’s Cognitive Examination III (ACE-III) and CANTAB Paired Associates Learning. Medial temporal lobe subregion volumes were automatically measured using high-resolution 3T MRI. The ACE-III was repeated after 12 months to assess the change in cognitive ability. We used univariate linear regressions and ROC curves to assess the ability of tests of delayed recall to predict cognitive decline on ACE-III over the 12 months.

**Results:**

Fifteen of the 46 participants declined over the year (≥ 3 points lost on ACE-III). Four-week verbal memory predicted cognitive decline in healthy older people better than clinical gold standard memory tests and hippocampal MRI. The best single-test predictor of cognitive decline was the 4-week delayed recall on the world list (*R*^2^ = .123, *p* = .018, *β* = .418). Combined with hippocampal subfield volumetry, 4-week verbal recall identifies those at risk of cognitive decline with 93% sensitivity and 86% specificity (AUC = .918, *p* < .0001).

**Conclusions:**

We show that a test of accelerated long-term forgetting over 4 weeks can predict cognitive decline in healthy older people where traditional tests of delayed recall cannot. Accelerated long-term forgetting is a sensitive, easy-to-test predictor of cognitive decline in healthy older people. Used alone or with hippocampal MRI, accelerated forgetting probes functionally relevant Alzheimer’s-related change. Accelerated forgetting will identify early-stage impairment, helping to target more invasive and expensive molecular biomarker testing.

## Background

Identifying and treating neuropathology leading to Alzheimer’s disease (AD) dementia in the early stages, when symptoms are minimal, is the optimal strategy to maintain quality of life. Developing effective treatments for AD may have been hampered by a failure to detect disease before pathological decline becomes inexorable [1]. Predicting progression at the stage of mild cognitive impairment (MCI) has had some success [2, 3]. However, in order to identify patients early, we need a marker that can be easily applied at scale with low cost and low patient burden at a stage before patients have symptoms that impair quality of life. If successful in trials, such a marker could be integrated into clinical care to enable early diagnosis and treatment.

Long-term memory over days or weeks may be an effective early marker of AD. Delayed recall over 1 week on a story task has been shown to have a much greater ability to distinguish healthy controls from those with MCI than recall over 30 min [4]. Another study showed that a delay of 6 weeks on story and complex figure recall tasks could identify significant impairment in a group of people who report ‘subjective’ memory impairment but who have no objective memory deficit on standard cognitive tests including delayed recall after 30 min [5]. This highlights that people with ‘subjective’ memory impairment may, in some cases, simply have impairment for which standard tests lack sufficient sensitivity. In a presymptomatic autosomal dominant AD cohort, Weston et al. [6] demonstrate that long-term memory on a verbal recall test over a 1-week delay not only highlights significant impairment but also predicts years-to-expected-age-of-dementia-onset. Finally, 1-week verbal memory is also impaired in otherwise asymptomatic APOE ε4 homozygotes compared to people with one or no ε4 alleles [7].

These longer-term memory tests may tax memory circuit functionality more than 30-min delayed recall tests—the current clinical gold standard cognitive task for detecting episodic memory problems which could be an early clinical manifestation of incipient AD. This ‘accelerated long-term forgetting’ (ALF) phenotype may represent a failure of memory consolidation processes [8] whereby information can be retained for short periods of time, but a successful conversion of labile short-term memory traces to permanent long-term memory is impossible. Such processes are thought to be primarily governed by the medial temporal lobe (MTL) [9–12], within which ALF may enable earlier identification of dysfunction.

The memory impairment observed in the earliest signs of AD can be attributed to damage to the hippocampus and MTL cortices. MTL atrophy, measured using magnetic resonance imaging (MRI), is a non-invasive early marker often in routine clinical use [13]. MTL areas affected early in AD are often reduced in volume even prior to the onset of clinically detectable cognitive symptoms [14].

In the present study, we aimed to test whether delayed recall over 4 weeks is a more sensitive early marker of AD-related cognitive decline than traditional tests of memory over 30 min. We also explored whether MTL subfield volume could complement ALF in identifying early signs of AD.

## Methods

### Participants

We recruited 53 cognitively healthy community-dwelling older participants with Addenbrooke’s Cognitive Examination III (ACE-III) scores of > 88, the upper threshold for detecting mild cognitive impairment [15]. Forty-six returned for follow-up 1 year later (mean age 70.7 ± 7.97). Twenty-one (46%) of these participants were female, and participants had 16.2 ± 3.52 years of education. Participants were recruited from local volunteer databases, the Join Dementia Research platform and word of mouth. All participants were verbally screened for a history of neurological disorders and memory problems in a telephone interview. All patients provided informed written consent prior to testing. Ethical approval was given by Frenchay NHS Research Ethics Committee.

### Cognitive testing

Cognitive change over the year was assessed using the Addenbrooke’s Cognitive Examination III (ΔACE-III), a freely available 15-min routine test for global cognition with high reliability and utility in dementia diagnosis [15–17]. The ACE-III comprises tests for 5 cognitive domains: attention, memory, language, fluency and visuospatial, and is scored out of a maximum of 100. Multiple versions of the ACE-III are available to allow for retesting. The version assigned to each participant at their baseline visit (either A or B) was randomized. The remaining version was administered at follow-up.

Delayed recall over 30 min and 4 weeks was tested on three separate tests:

1. Word list—a 16-word list based on the California Verbal Learning Task-II (CVLT-II) [18]; maximum score, 16
2. Story—from the Rivermead Behavioural Memory Test-3 (RBMT-3) [19]; maximum score, 21
3. Complex figure—based on the Rey-Osterrieth Complex Figure Task (RCFT) [20]; maximum score, 36

These tests were adapted from their standard protocols (i.e. that of CVLT-II, RBMT-3 or RCFT) in that participants were trained to 75% accuracy on each test, with 2–5 learning trials to equate learning between individuals [21]. Standard CVLT-II procedure involves exactly 5 learning trials, and standard RBMT-3 story protocol involves just one, regardless of performance. Similarly, the RCFT procedure usually incorporates just one copy and one recall test during the learning phase. For the adapted complex figure task, copy and recall trials were both repeated 2–5 times throughout the learning phase. Both delayed recall time points were performed in person, and participants were not told that recall would be tested after 4 weeks.

We took a pragmatic approach in this paper to try and identify the ideal test which would be quick and easy to administer and provide high sensitivity and specificity. Although a single test would be ideal, as it would be feasible to do all 3 of these tests in clinical practice we calculated the composite score (by averaging the scores as a proportion of the maximum possible score for each test) to see if we could increase the sensitivity and specificity at any given testing time point for cognitive decline over a year. A priori (based on data generated by others), we suspected that either word list memory or story memory over 4 weeks would be most sensitive to AD-related cognitive change.

Further testing included the Paired Associate Learning (PAL) using CANTABeclipse v5 (Cambridge Cognition Ltd.; Cambridge, UK). This test has demonstrated utility in detecting cognitive deficit due to AD [22–25], so we included it to compare whether our tests could better predict cognitive decline. However, we acknowledge this test is not without its criticisms and limitations [26, 27]. Measures used included total accuracy, mean reaction time and maximum level reached.

### Imaging parameters

All MRI scans were acquired on a Siemens Magnetom Skyra 3T system. The volumetric imaging protocol included a 3D T1-weighted MPRAGE (acquired resolution 0.86 mm isotropic) and two 2D T2-weighted high-resolution hippocampal turbo spin-echo sequences (multi-contrast and single-contrast) (reconstructed resolution 0.34 × 0.34 × 1.5 mm). Full imaging details are as described by Nurdal et al. [28].

### Image analysis

**Fig. 1:**
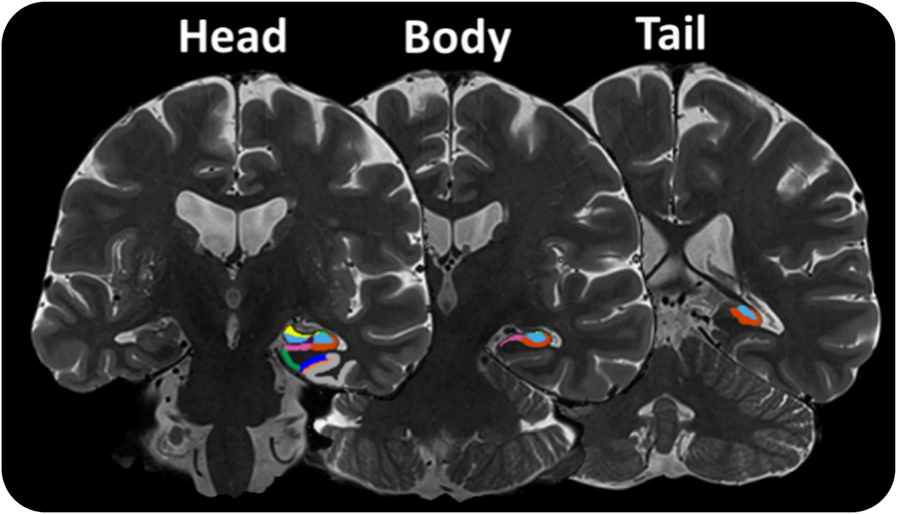
MTL subfield mask example. Three skull-stripped coronal sections of summed-over-echoes T2-w scan of a single participant are shown with anterior MTL (head) on the left and posterior hippocampus (tail) on the right. Shown are subfields CA1 (red), CA2 (light green), CA3 (yellow), DG (light blue), EC (dark green), BA35 (dark blue) and BA36 (grey)

MTL subfields were demarcated using the automated hippocampal subfield segmentation (ASHS) software (rev103), using the UPENN atlas comprising a mixture of older adults and MCI patients [29]. An example of ASHS output is shown in Fig. 1. All hippocampal masks created as an output of ASHS were visually inspected for quality. In cases where the multi-echo image was either not present or of too poor quality due to movement artefacts, the single-echo TSE was used instead. We have shown in-house that ASHS outputs from either scan type are not significantly different from one another. Three participants were excluded from the volumetric analysis due to poor mask quality. Analysis of individual subfields was performed on CA1, dentate gyrus (DG), subiculum (SUB), entorhinal cortex (EC) and perirhinal cortex (PC). PC was defined as the BA35 label in the UPENN atlas. CA2 and CA3 were included in models combining all subfields. Whole hippocampal volume (CA1–3 + DG + SUB) was also analysed, to highlight the added value of subfield analyses. Volumes were all normalized to intracranial volume.

### Statistical analyses

#### Main analyses

Predictive power of delayed recall scores was performed in two ways: continuous and discrete (‘did they decline or not?’). For the former ‘continuous’ analysis, univariate linear regressions with delayed recall score of a given test as the independent variable and ΔACE-III as the outcome variable. For the latter ‘discrete’ analysis, participants were split into two groups: those whose ACE-III scores declined over the year vs those whose scores did not decline.

This threshold was defined by the reliable change index (RCI) [30], which is calculated using the following formulae:

$$ \mathrm{SE}={\mathrm{SD}}_{\mathrm{norm}}\times \sqrt{2}\times \sqrt{1-r} $$

$$ \mathrm{RCI}=\mathrm{SE}\times 1.96 $$

where SE is the standard error of change on a test, SD_norm_ is the standard deviation of ACE-III score in the normative data (2.7) [15] and *r* is the reliability of the test, in this case, given as Cronbach’s alpha (0.88) [16]. The resulting reliable change index is 2.59. Therefore, a decline of 3 or more points was deemed representative of mild but statistically significant cognitive decline.

We used receiver operating characteristic (ROC) curves to assess the predictive capability of each variable in identifying those who declined vs those who did not. Predicted probabilities from multiple logistic regressions were used to create ROC curves of multiple combined variables. ROC Curves were assessed using the area under the curve (AUC) and compared using DeLong’s test for dependent ROC curve comparisons [31]. Standard errors (SE) for AUC are shown in the supplementary information (Supplementary Table 2).

#### Exploratory analysis

In order to fully assess the added value of MRI volumetry in predicting cognitive decline, we calculated ROC AUC for various combinations of variables (Supplementary Table 2). In order to explore the relationships between different tests of delayed recall, we also calculated Pearson’s correlation coefficients for each pairing (supplementary Table 1).

All reported *p* values are two-tailed (*α* = 0.05), and uncorrected for multiple comparisons given that tests were not fully independent from one another (supplementary Table 1). However, Bonferroni-corrected *p* values (corrected across all 3 delayed recall tests) are shown where appropriate for full transparency (as *p*_bonferroni_). All analyses were performed in IBM SPSS Statistics 24, except DeLong tests to compare ROC curves which were carried out in MedCalc v19.4.

## Results

**Fig. 2:**
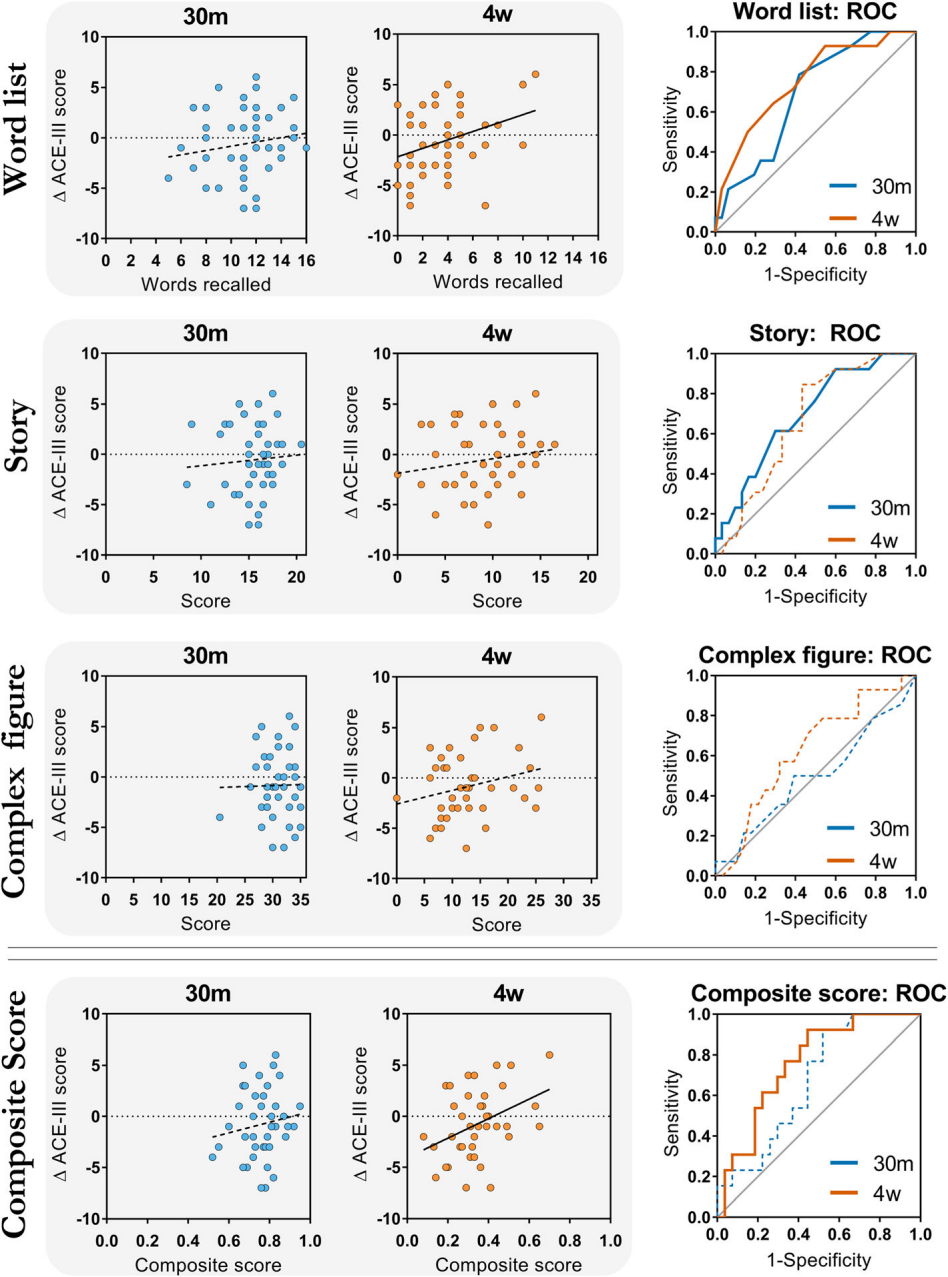
Delayed recall predicting cognitive decline over 1 year: linear regressions (left) and ROC curves (right). Linear regression panels (left) show raw data for each test and ΔACE-III. Smaller scores on a 4-week word list recall indicate a more negative score change. Solid lines represent statistically significant regressions/ROC curves (*p* < .05). Dotted lines represent non-significant regressions/ROC curves (*p* > .05). 30m, 30-min recall scores; 4w, 4-week recall scores; ACE-III, Addenbrooke’s Cognitive Examination III

Four-week (4w) recall on the word list task predicted ΔACE-III (*R*^2^ = .123, *p* = .018; *p*_bonferroni_ = .054) (Table 1; Fig. 2). There was no such relationship between ΔACE-III and 30-min (30m) recall on the word list (*R*^2^ = .028, *p* = .274; *p*_bonferroni_ = .822). Scores at either time point of the story (30m: *R*^2^ = .006, *p* = .629; 4w *R*^2^ = .027, *p* = .289) and complex figure tasks (30m: *R*^2^ = .0003, *p* = .912; 4w: *R*^2^ = .045, *p* = .171) could not predict ΔACE-III. Strikingly, in this healthy cohort, neither whole hippocampal volume nor volume of any single hippocampal subfield or MTL cortical region was correlated with change in performance over the year (Table 1). Furthermore, PAL measures did not predict cognitive decline.

**Table 1:** Univariate linear regression statistics for delayed recall, PAL scores and MTL region volumes predicting ΔACE-III

Predictors	*β* (standardized)	*R*^2^	*p*	Number
Word List				
30m	.167	.028	.274	45
4w	.350	.123	.018	45
Story				
30m	.076	.006	.629	43
4w	.165	.027	.289	43
Complex figure				
30m	.018	.0003	.912	42
4w	.246	.061	.116	42
PAL				
Accuracy	.194	.037	.225	41
Reaction time	− .021	.0004	.898	41
Max level	.064	.004	.689	41
Whole hippocampus	− .072	.005	.646	43
CA1	− .084	.007	.590	43
DG	− .059	.003	.707	43
SUB	.005	< .0001	.976	43
EC	.098	.010	.532	43
PC	.173	.030	.269	43

The 4w composite score significantly positively predicted ΔACE-III (*R*^2^ = .152, *p* = .013, *β* = .390), yet 30m composite recall score did not (*R*^2^ = .021, *p* = .378, *β* = .143) (Fig. 2).

Fifteen of the 46 people in this cohort declined more than expected using the reliable change index. Demographic and delayed recall data for decliners and non-decliners are shown in Table 2. Raw cognitive test data for each can be found in supplementary information.

**Table 2:** Demographic information and delayed recall data for people who did or did not decline over the year

	Non-decliner	Decliner	Total
*N* (male:female)	31 (16:15)	15 (9:6)	46 (25:21)
Age (years)	71.1 ± 8.92	69.9 ± 5.74	70.7 ± 7.97
YOE	16.7 ± 3.41	15.1 ± 3.61	16.2 ± 3.35
ACE-III /100			
Baseline	95.0 ± 3.03	94.7 ± 3.20	94.9 ± 3.05
Follow-up	96.1 ± 2.73	90.5 ± 3.14	94.2 ± 3.88
Word list /16			
30m	11.5 ± 2.55	9.73 ± 2.37	10.9 ± 2.61
4w	4.35 ± 2.79	2.14 ± 2.03	3.67 ± 2.76
Story /21			
30m	16.0 ± 2.22	14.2 ± 2.49	15.4 ± 2.42
4w	9.70 ± 4.07	7.65 ± 2.85	9.08 ± 3.83
Complex figure /36			
30m	31.0 ± 2.57	30.2 ± 4.07	30.7 ± 3.15
4w	13.5 ± 6.42	11.5 ± 4.95	12.9 ± 5.99

Data are shown as mean ± standard deviation. Maximum scores for each cognitive test are shown in the left column

*YOE* years of education, *30m* 30-min delayed recall time point, *4w* 4-week delayed recall time point

Both 30m and 4w word list recall scores distinguished decliners from non-decliners. In line with our hypothesis, the 4w recall time point had the strongest discriminatory power for a single variable with an AUC of .752 (SE = .078, *p* = .007). 30m verbal recall (AUC = .687, SE = .080 *p* = .047), and 30m (story: AUC = .699, SE = .083, *p* = .040; complex figure: AUC = .497, SE = .099, *p* = .979) and 4w measures (story: AUC = .671, SE = .083, *p* = .079; complex figure: AUC = .625, SE = .090, *p* = .191) in other tests had weaker discriminatory power. The 4w composite recall score was able to predict ACE-III decline status (AUC = .761, SE = .076, *p* = .008), whereas the 30m composite score was less effective (AUC = .674, SE = .085, *p* = .078). Comparing the ROC curves using the Delong method [31] did not reveal statistically significant differences between 30m and 4w AUCs for any test (word list: *p* = .472; story: *p* = .785; complex figure: *p* = .437; composite: *p* = .369).

**Fig. 3:**
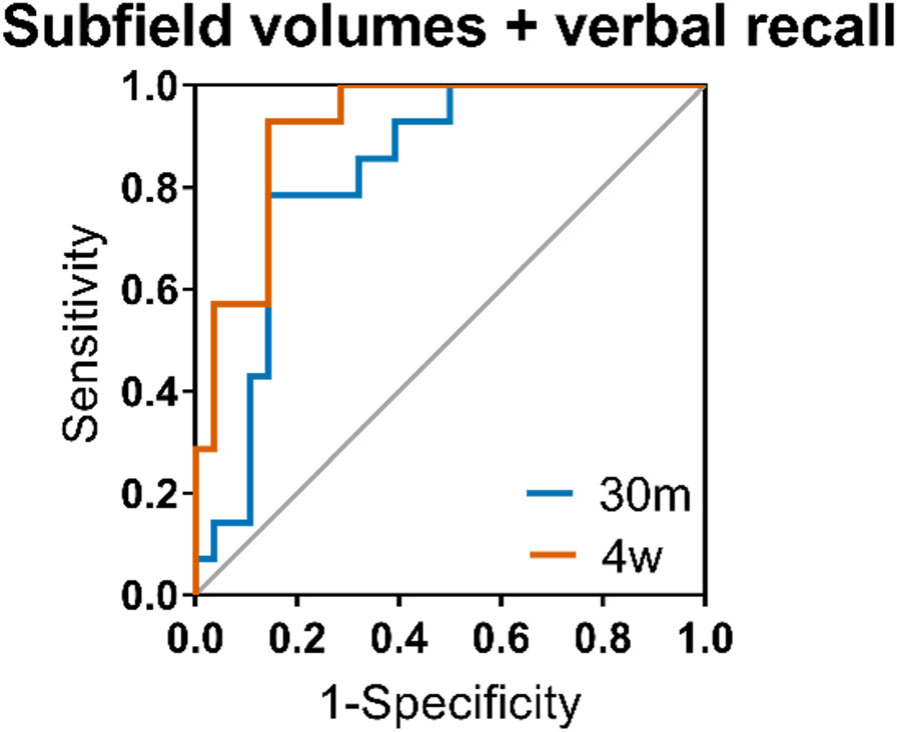
Accuracy of delayed verbal recall and MTL subfield volumes predicting cognitive decline. Shown are receiver operating characteristic (ROC) curves for individual combined subfields (CA1–3, DG, EC, BA35) and either 30-min (30m) or 4-week (4w) recall on the word list

Combining memory test scores with MTL regional volumes improves classification. 4w word list recall combined with individual subfield volumes produced the best single-test model with an AUC of .918 (*p* < .0001; Fig. 3). The optimal cut-off has 93% sensitivity and 86% specificity. The equivalent model using 30m word list recall score achieved an AUC of .829 (*p* = .001). In comparison, MTL subfields alone produced a predictive model with an AUC of .802 (*p* = .001). All ROC data can be seen in supplementary Table 2.

## Discussion

We report that extending the delay period over which recall, particularly verbal recall, is tested from 30 min to 4 weeks can markedly improve the identification of people likely to decline cognitively in the coming year. This is the first time this has been shown in a longitudinal cohort study. ALF improves classification accuracy beyond that which is achieved using MTL subfield volumes. These findings support the clinical utility of much longer-term memory tests in identifying the very subtle neuropathology that occurs years before a diagnosis of AD dementia [32], though further research is required to fully validate their use in predicting conversion to dementia.

Previous work in a selective presymptomatic cohort with the extremely rare autosomal dominant form of Alzheimer’s disease suggested that ALF might be a sensitive marker of early change despite having normal scores after a 30-min delay [6]. They tested all three of a story, word list and complex figure task over a delay of 1 week, and only on the word list was longer-term memory worse in genetically at-risk participants. Here, we utilized a real-world sample to reveal that long-term word list memory has the potential to be applied to the general older population as a marker of future cognitive decline. These complementary findings suggest that the word list may present the best single test to predict at-risk patient groups and that story learning or visuospatial memory tasks may be less sensitive. Alignment of our results with those from a different study in a cohort genetically at-risk is encouraging as to the reliability of this finding. Future clinical trials could utilize memory tests over a longer time frame as a sensitive marker of change. It should be noted that the 4-week verbal recall test predicting cognitive change over the year was not below the *α* threshold of statistical significance after a Bonferroni correction (*p* = .054) across the 3 cognitive tests used. However, the Bonferroni method is a highly conservative method of correcting for multiple comparisons, and given that the three tests cannot be regarded as fully independent (as shown in supplementary Table 1), we believe this still to be a robust, albeit preliminary, finding. Future work should aim to confirm or deny this finding.

ROC curves for word list recall show that very high levels of sensitivity are achievable with small trade-offs in specificity. For example, a cut-off score of 4.5 at the 4w time point allows 93% of people who decline by more than three points on ACE-III to be detected, even though 55% of people below this cut-off do not decline by more than this amount. This enables the test to be used as a wide-net screening tool, the main goal of which is to reduce the number of people having more expensive and invasive testing for probable AD, such as CSF analyses or amyloid/tau PET scans [33]. A 4-week memory task takes only a few minutes to administer and could transform standard clinical neuropsychology testing (where delayed recall of a word list over 30 min is usually administered). This test could even be adapted to be performed over the phone or delivered by a mobile app, to reduce face-to-face clinic visits. There is the caveat of having to wait 4 weeks to get any result from this test. However, although we used a 4-week delay paradigm in this study, we do not show that it is necessary to wait this long to measure ALF. Indeed, shorter delays are less susceptible to confounding from the variation in events between delay time points. The delay period in past work ranges from 1 to 6 weeks [4–6], but further work is required to determine the optimal delay that is sensitive to cognitive dysfunction, but least burdensome for participants and clinicians.

Given the critical role for MTL in long-term memory consolidation [34], ALF may reflect subtle MTL damage, such as that which occurs in the prodromal stages of AD. In combination with MTL subregion volumes, 4-week verbal recall scores predict cognitive decline in cognitively healthy individuals with sensitivity and specificity similar to that of much more expensive molecular biomarkers tests (PET scanning and CSF analysis) [35–37]. The present analyses provide further justification for the incorporation of MRI and automated volumetry techniques in clinical diagnostic processes [13, 38, 39]. As neuroimaging, often MRI, is part of routine clinical screening processes for neurological disease, the combination of cognitive tests with volumetry is highly practical and easily translatable. It is interesting to note that although MTL volumes are able to predict the presence or absence of cognitive decline (in our ROC analysis), they were very poor in predicting any linear association with cognitive change. This may indicate a non-linear relationship between the two, whereby only MTL values below a certain threshold are indicative of cognitive decline, and cognitive change in people with larger MTL volumes is more determined by other factors (such as tissue damage that has not yet resulted in volume change detectable on MRI [40, 41]). This supports theories of graceful degradation and cognitive reserve [42] and should be further explored in studies with larger sample sizes. ALF may also require the functionality of networks beyond the MTL that are still affected in early AD, such as those involving the thalamus [43].

It could be suggested that the observed ALF phenotype is explained by the differences in practice effects between individuals. Lack of practice effects (failure to improve performance when a task is repeated) has been shown to be predictive of cognitive decline in people with amnestic mild cognitive impairment [44]. This seems relatively unlikely to be the case here as task instructions requirements were conceptually very simple—free recall tests, rather than re-administration of any learning materials. Furthermore, during the learning phase, differences in individual learning rates were controlled for by including the 75% accuracy threshold, above which learning stimuli were not repeated. We therefore believe an attenuated practice effect to be a distinct measure, albeit potentially with similar neurobiological underpinnings, whereby impaired memory consolidation circuits may impair an individual’s ability to benefit from repeated trials.

### Limitations

A limitation of the present study is that the global cognitive decline seen over the year cannot be directly attributed to AD without assessment of amyloid and/or tau status via CSF or PET. This was not collected as part of the present study but is an area of future interest and presents an obvious next step for this research. Despite this, we suggest that tests of delayed recall may predict cognitive decline that is specifically due to AD pathology given the role of MTL in long-term memory consolidation [34, 45] and the susceptibility of MTL to early AD pathology. Furthermore, as discussed, this study is highly in line with the findings of Weston et al. [6], which directly links delayed recall over days/weeks to autosomal dominant AD.

Although the 4-week recall time point of the word list task produced the greatest AUC for any single cognitive test in detecting which people experienced cognitive decline, the differences between time points were relatively small. This is in part due to the high correlations between test time points, at least for the story and word list (supplementary Table 1); however, this is also due to our relatively small sample size. A larger sample size would be required to confirm that differences in classification ability are statistically significant, as well as to improve the certainty of conclusions from all conducted analyses. Future studies may use our findings to assist in calculating sample sizes required for sufficient power to detect such statistical differences.

Throughout this paper, we discuss mild cognitive decline defined as a statistically reliable change in score on the ACE-III, a test of general cognitive function. Although a drop of just 3 points is statistically significant, it may not be clinically significant in terms of a functional decline and is certainly not necessarily indicative of a decline to a level of MCI. Given the group (healthy older adults), sample size (46) and follow-up period of this study (1 year), a subtle measure of cognitive change was necessary, as opposed to measuring conversion to MCI or AD. We infer that a subtle cognitive decline over this timescale may indicate further cognitive decline over longer timescales. In this regard, the marker does not necessarily need to be directly clinically functionally significant, but rather, it would herald the need for further investigation to detect the cause of the change and, where possible, avoid a functionally significant change. Further follow-ups of these participants would be necessary to observe which people decline further or even which people later receive diagnoses of dementia. Further research is also required to explicitly test the responsiveness of the ACE-III to detecting subtle cognitive decline.

Another limitation of our study is the validity of the cognitive tests used to measure delayed recall. Although based on well-validated tasks designed to measure recall at 30 min, we modified the protocols slightly (e.g. by including a learning criterion and varying the number of learning trials). In doing so, there was a risk that we invalidated the tests for measuring delayed recall at 30 min. Although the data are not comparable to normative data of the tests on which they are based, we believe they still provide robust measures of delayed recall at both time points as we observed substantial variation in scores at both time points in all 3 tests, indicating a lack of floor and ceiling effects.

Finally, it could be argued that an incorporation bias exists, given that the cohort was defined as healthy by having an ACE-III > 88, 26 points of which are achieved from tests of memory. We do not believe this to be a factor which biases our results, as memory is tested over delays of no more than 10 min during the ACE-III, and this forms a small part of the entire ACE-III score (the 10-min delayed recall test is worth just 7 points in total). Even if the cohort was defined solely on having normal 30-min recall at baseline, this could not be expected to influence whether 30-min or 4-week memory would better predict cognitive decline over the year.

## Conclusions

In summary, we show that a test of accelerated long-term forgetting over 4 weeks can predict cognitive decline in healthy older people where traditional tests of delayed recall cannot. This highlights its potential in screening patients for clinical trials and practice either in conjunction with volumetry or as a standalone test. This could reduce the number of patients who need expensive biomarker testing for Alzheimer’s disease, increasing the feasibility of large-scale early clinical trials. Further research is required to explore the relationship between ALF and molecular biomarker profiles to define the specificity of longer-term recall deficits to early AD pathology.

## Supplementary information

**Additional file 1.** Supplementary tables.

## Data Availability

The datasets used and/or analysed during the current study are available from the corresponding author on reasonable request.
