# Supplementary material for: Accelerated long-term forgetting in healthy older adults predicts cognitive decline over 1 year
Source: Alzheimers Res Ther. 2020 Sep 28;12:119. doi: 10.1186/s13195-020-00693-4 (PMC7523317; doi:10.1186/s13195-020-00693-4)
Supplement: Supplementary file 1 — Additional file 1. Supplementary tables. [file 13195_2020_693_MOESM1_ESM.docx]

**Supplementary information**

Supplementary Table 1 | Correlation matrix showing linear relationships between delayed recall scores from different tests. Statistics shown are Pearson’s r and raw p-values α=0.05. 30m = 30-minute delayed recall timepoint; 4w = 4-week delayed recall timepoint.

|  |  |  | Word List | |  | Story | |  | Complex Figure | |
| --- | --- | --- | --- | --- | --- | --- | --- | --- | --- | --- |
|  |  |  | 30m | 4w |  | 30m | 4w |  | 30m | 4w |
| Word List | 30m | r | - | .402^**^ |  | .416^**^ | .176 |  | .377^*^ | .145 |
|  |  | p | - | .006 |  | .005 | .258 |  | .014 | .353 |
|  | 4w | r | .402^**^ | - |  | .306^*^ | .365^*^ |  | .148 | .492^**^ |
|  |  | p | .006 | - |  | .043 | .016 |  | .351 | .001 |
|  |  |  |  |  |  |  |  |  |  |  |
| Story | 30m | r | .416^**^ | .306^*^ |  | - | .392^**^ |  | .134 | .177 |
|  |  | p | .005 | .043 |  | - | .009 |  | .403 | .262 |
|  | 4w | r | .176 | .365^*^ |  | .392^**^ | - |  | -.217 | .486^**^ |
|  |  | p | .258 | .016 |  | .009 | - |  | .179 | .001 |
|  |  |  |  |  |  |  |  |  |  |  |
| Complex Figure | 30m | r | .377^*^ | .148 |  | .134 | -.217 |  | - | .186 |
|  |  | p | .014 | .351 |  | .403 | .179 |  | - | .238 |
|  | 4w | r | .145 | .492^**^ |  | .177 | .486^**^ |  | .186 | - |
|  |  | p | .353 | .001 |  | .262 | .001 |  | .238 | - |

Supplementary Table 2. ROC curve results for MTL volumes and delayed recall tests predicting presence of ACE-III decline.

| Predictor(s) | AUC | Std Error | p | n |
| --- | --- | --- | --- | --- |
| **MRI variables only** |  |  |  |  |
| Hipp | .510 | .107 | .919 | 43 |
| Hipp + EC + PC | .707 | .080 | .027 | 43 |
| All MTL subregions | .802 | .066 | .001 | 43 |
| **Word list** |  |  |  |  |
| 30m recall | .687 | .080 | .047 | 45 |
| + Hipp | .724 | .082 | .019 | 42 |
| + Hipp + EC + PC | .732 | .076 | .015 | 42 |
| + All MTL subregions | .829 | .064 | .001 | 42 |
| 4w recall | .752 | .078 | .007 | 45 |
| + Hipp | .742 | .081 | .011 | 42 |
| + Hipp + EC + PC | .829 | .063 | .001 | 42 |
| + All MTL subregions | .918 | .041 | <.0001 | 42 |
| **Story** |  |  |  |  |
| 30m recall | .699 | .083 | .040 | 43 |
| + Hipp | .752 | .084 | .011 | 40 |
| + Hipp + EC + PC | .758 | .077 | .009 | 40 |
| + All MTL subregions | .838 | .062 | .001 | 40 |
| 4w recall | .671 | .083 | .079 | 43 |
| + Hipp | .744 | .087 | .014 | 40 |
| + Hipp + EC + PC | .803 | .078 | .002 | 40 |
| + All MTL subregions | .869 | .063 | .0002 | 40 |
| **Complex Figure** |  |  |  |  |
| 30m recall | .497 | .099 | .979 | 42 |
| + Hipp | .531 | .102 | .747 | 39 |
| + Hipp + EC + PC | .666 | .087 | .089 | 39 |
| + All MTL subregions | .786 | .074 | .003 | 39 |
| 4w recall | .625 | .090 | .191 | 42 |
| + Hipp | .637 | .093 | .160 | 39 |
| + Hipp + EC + PC | .754 | .081 | .009 | 39 |
| + All MTL subregions | .837 | .067 | .001 | 39 |
| **Composite recall score** |  |  |  |  |
| 30m recall | .674 | .085 | .078 | 40 |
| + Hipp | .766 | .086 | .008 | 37 |
| + Hipp + EC + PC | .779 | .077 | .006 | 37 |
| + All MTL subregions | .824 | .070 | .001 | 37 |
| 4w recall | .761 | .076 | .008 | 40 |
| + Hipp | .817 | .080 | .002 | 37 |
| + Hipp + EC + PC | .894 | .056 | <.0001 | 37 |
| + All MTL subregions | .920 | .044 | <.0001 | 37 |

AUC = Area under the ROC Curve. Hipp = Hippocampal volume. N=sample size of each statistical test.
